# A New Class of Wheat Gliadin Genes and Proteins

**DOI:** 10.1371/journal.pone.0052139

**Published:** 2012-12-20

**Authors:** Olin D. Anderson, Lingli Dong, Naxin Huo, Yong Q. Gu

**Affiliations:** 1 Genomics and Gene Discovery Research Unit, Western Regional Research Center, Agricultural Research Service, United States Department of Agriculture, Albany, California, United States of America; 2 Department of Plant Sciences, University of California Davis, Davis, California, United States of America; 3 The Key Laboratory of Plant Cell and Chromosome Engineering, Institute of Genetics and Developmental Biology, Chinese Academy of Sciences, Beijing, China; Ben-Gurion University, Israel

## Abstract

The utility of mining DNA sequence data to understand the structure and expression of cereal prolamin genes is demonstrated by the identification of a new class of wheat prolamins. This previously unrecognized wheat prolamin class, given the name δ-gliadins, is the most direct ortholog of barley γ3-hordeins. Phylogenetic analysis shows that the orthologous δ-gliadins and γ3-hordeins form a distinct prolamin branch that existed separate from the γ-gliadins and γ-hordeins in an ancestral Triticeae prior to the branching of wheat and barley. The expressed δ-gliadins are encoded by a single gene in each of the hexaploid wheat genomes. This single δ-gliadin/γ3-hordein ortholog may be a general feature of the Triticeae tribe since examination of ESTs from three barley cultivars also confirms a single γ3-hordein gene. Analysis of ESTs and cDNAs shows that the genes are expressed in at least five hexaploid wheat cultivars in addition to diploids *Triticum monococcum* and *Aegilops tauschii*. The latter two sequences also allow assignment of the δ-gliadin genes to the A and D genomes, respectively, with the third sequence type assumed to be from the B genome. Two wheat cultivars for which there are sufficient ESTs show different patterns of expression, i.e., with cv Chinese Spring expressing the genes from the A and B genomes, while cv Recital has ESTs from the A and D genomes. Genomic sequences of Chinese Spring show that the D genome gene is inactivated by tandem premature stop codons. A fourth δ-gliadin sequence occurs in the D genome of both Chinese Spring and *Ae. tauschii*, but no ESTs match this sequence and limited genomic sequences indicates a pseudogene containing frame shifts and premature stop codons. Sequencing of BACs covering a 3 Mb region from *Ae. tauschii* locates the δ-gliadin gene to the complex *Gli-1* plus *Glu-3* region on chromosome 1.

## Introduction

The γ-type seed prolamins are widely distributed within the Triticeae, have been studied most extensively in wheat (γ-gliadins), barley (γ-hordeins), and rye (γ-secalins), and have been proposed to be the most ancestral of the Triticeae prolamins [1]. The wheat γ-gliadins are estimated of to be encoded by 15–40 genes [2], and there are some 200 γ-gliadin sequences in Genbank for *Triticum aestivum* (bread wheat) plus more from other *Triticum* species and Triticeae genera. The barley γ-hordeins are not as well studied, but have been tentatively separated into γ1, γ2, and γ3 classes based on limited data from electrophoretic mobility of barley seed proteins, N-terminal sequences, and antibody specificity [3], [4]. However, there are relatively few gene sequences for barley γ-hordeins in Genbank; e.g., only two *Hordeum vulgare* γ3-hordein sequences – one covering a complete coding region (AK251750, [5]) and a partial sequence (X72628, [6]) along with 21 partial or complete more divergent *H. chilense* coding sequences [7]. Both γ1 and γ2 barley probes of Genbank return the same three matches (X13508 [8], M36378 [8], and AJ580585 [9]: M36378 and X13508 are the same sequence. The reports and Genbank entries assign AJ580585 as a γ2-hordein and M36378 as a γ1-hordein. The original classification was initially based on factors which have only a potential relationship to evolutionary connection of gene sequences and are not definitive. It has also previous been proposed that the γ1- and γ2-hordeins are more similar to each other than they are to γ3-hordein [3], [4]. Previously, comparisons have indicated a orthologous relationship between the wheat γ-gliadins and barley γ1- and γ2-hordeins [3], but no closely related wheat sequence to γ3-hordein has been reported.

Since the prolamins of wheat are largely responsible for the visco-elastic properties of wheat doughs [10], and as such the basis for the economic and agronomic importance of wheat, as complete an understanding of the wheat seed storage protein complement is important. In addition, the Triticeae prolamins are associated with celiac disease – an autoimmune disorder triggered by exposure to epitopes common in prolamins [11]. One proposed strategy has been to eliminate the causative classes of prolamins, such as the γ-gliadins, by either breeding or genetic engineering by homology-related gene silencing which has been used to reduced γ-gliadin synthesis [12]. For such strategies to be maximally successful, it is again necessary to have as complete an understanding as possible of the variety and composition of the different wheat prolamin classes.

As more genomic and EST sequences become available for the Triticeae species, these resources can be used to investigate prolamin gene family structure, and can allow discovery of genes and diversity missed in directed studies. In the present report, an examination of sequences in Genbank and next-generation high-throughput sequences of hexaploid wheat and a diploid wheat ancestor revealed that a previously unrecognized wheat gene and storage protein orthologous to the barley γ3-hordein exists and is evolutionarily distinct enough from other γ-type gliadins sequences to be considered a separate class of wheat prolamins. This separate class, with the proposed designation of δ-gliadins, is shown to be encoded by a single active gene in each of the hexaploid wheat genomes and diploid wheat *Aegilops tauschii,* and a single orthologous γ3-hordein gene in barley. Sequencing of *Ae. tauschii* BAC contigs finds the new gliadin gene to be part of the complex *Gli-1*/*Glu-3* region of the wheat genome known to contain genes for γ- and ω-gliadins and LMW-glutenins.

## Materials and Methods

### Mining Sequence Databases for Triticeae Prolamins

Sequences related to the γ-type prolamins were identified using the blast facilities at NCBI (www.ncbi.nlm.nih.gov). Triticeae genomic and cDNA sequences were retrieved from Genbank at NCBI, and EST sequences were retrieved either from Genbank or from the GrainGenes wEST site (http://wheat.pw.usda.gov/wEST/blast/) which allows blasting individual wheat and barley cultivar EST collections.

Coding regions of prolamin genes were used to search Genbank for ESTs by blastn. Minimal expectation values were determined empirically for each search. Assignments to specific prolamin classes were confirmed by assembling EST sequences with examples of all relevant prolamin classes. For example, a blastn search of wheat ESTs used a minimal expectation value of e^−30^. These ESTs were assembled with examples of α-, γ-, and ω-gliadins along with LMW glutenins and δ-gliadins (described in Results and Discussion) consensus sequences. Confirmation of the EST belonging to the δ-gliadin class was if the EST assembled with the δ-gliadins and not other classes such as the γ-gliadins. A similar procedure was used for barley and unambiguously assigned relevant barley ESTs to either the γ1- plus γ2-hordein contig or the separate γ3-hordein contig.

### Chinese Spring Hexaploid Genomic DNA Sequences

A 5×454 sequence read resource, including blast facility, for wheat cv Chinese Spring is available at http://www.cerealsdb.uk.net/and described in Brenchley et al. [13]. Prolamin probes identified matching 454 reads which were downloaded, assembled with the Seqman module of the Lasergene suite (DNAstar, Inc.), and manually separated into distinguishable read sets which were then reassembled. Average 454 read lengths are 384 bp. After discarding reads shorter than 100 bp, the average read utilized was 450 bp. Extensions of unique sequences were carried out by reiterative probing of the Chinese Spring 454 reads, reassembling, and then removing mismatching reads. Final sequences were the consensus of overlapping multiple 454 reads. Chinese Spring ESTs confirmed the 454 sequence over the range covered by ESTs, and 454 extension beyond available EST matching sequences were required to include at least two independent 454 reads with 100% matching sequences. The reported consensus sequences were terminated when this criterion was not met. All consensus DNA sequences and derived amino acid sequences for this report are found in (File S1).

### *Aegilops tauschii* Diploid Genomics Sequences

To generate shotgun sequence reads of the *Ae. tauschii* genome, preparation and sequencing of the 454 sequencing libraries were made according to the manufacturer’s instructions (GS FLX Titanium General Library preparation kit/emPCRkit sequencing kit, Roche Diagnostics, http://www.roche.com). Briefly, ten µg of *Ae. tauschii* accession AL78/8 genomic DNA was sheared by nebulization and fractionated with agarose gel electrophoresis to isolate 400–750 bp fragments and the sized fragments used to construct a single-stranded shotgun library. The library was quantified by fluorometry using Quant-iT RiboGreen reagent, and processed by emulsion PCR amplification. The library was sequenced with GS FLX Titanium following manufacturer recommendations (Roche Diagnostics, http://wheat.pw.usda.gov). The raw sequencing data from 454 instrument were processed using Roche gsAssembler ver2.6. The ssf file containing the sequence data with quality score for each base were generated for each Roche 454 run and used for contig assembly with the gsAssembler. Raw *Ae. tauschii* 454 reads and assemblies can be blasted and sequences downloaded at http://avena.pw.usda.gov/RHmapping/blast2/- part of the GrainGenes (http://wheat.pw.usda.gov) suite of databases and services. Probing for prolamin reads and assembling reads was as described above for Chinese Spring.

### BAC Contigs Assembly and Sequencing

The BAC clones harboring wheat prolamin genes were obtained by screening a *Ae. tauschii* BAC library using wheat gliadin/LMW-GS probes using protocols previously described [14]. The clone IDs of those positive BAC clones were used to search corresponding BAC contigs in the *Ae. tauschii* physical mapping project (http://probes.pw.usda.gov:8080/wheatdb/). Two BAC contigs, designated Ctg10 and Ctg14, were identified. A total of 28 BAC clones representing the minimum tilling path (MTP) of the BAC contigs were selected for sequencing with Roche 454. An average of five overlapping BAC clones were pooled and sequenced to ∼ 20× coverage. In addition, a 3-kb paired library for these BAC clones were made and sequenced to 10× coverage. These sequenced data were used for sequence assembly. The contigs obtained from the *de novo* assembly were ordered by paired-end reads to form scaffolds. Scaffolds were further oriented by mapping BAC end sequences (BES) based on the known physical map MTP order. Contig sequences were submitted to Genbank as accession JX295577.

### Sequence Analysis

DNA and protein sequence analysis used mainly the Lasergene (DNAStar, Inc.) Editseq, Megalign, and Seqman modules. Additional resources included blast facilities at NCBI, Gramene (http://gramene.org), CerealsDB (http://cerealsdb.uk.net), GrainGenes (http://wheat.pw.usda.gov/wEST), and the *Ae. tauschii* physical mapping project (probes.pw.usda.gov:8080/wheatdb).

## Results and Discussion

**Figure 1:**
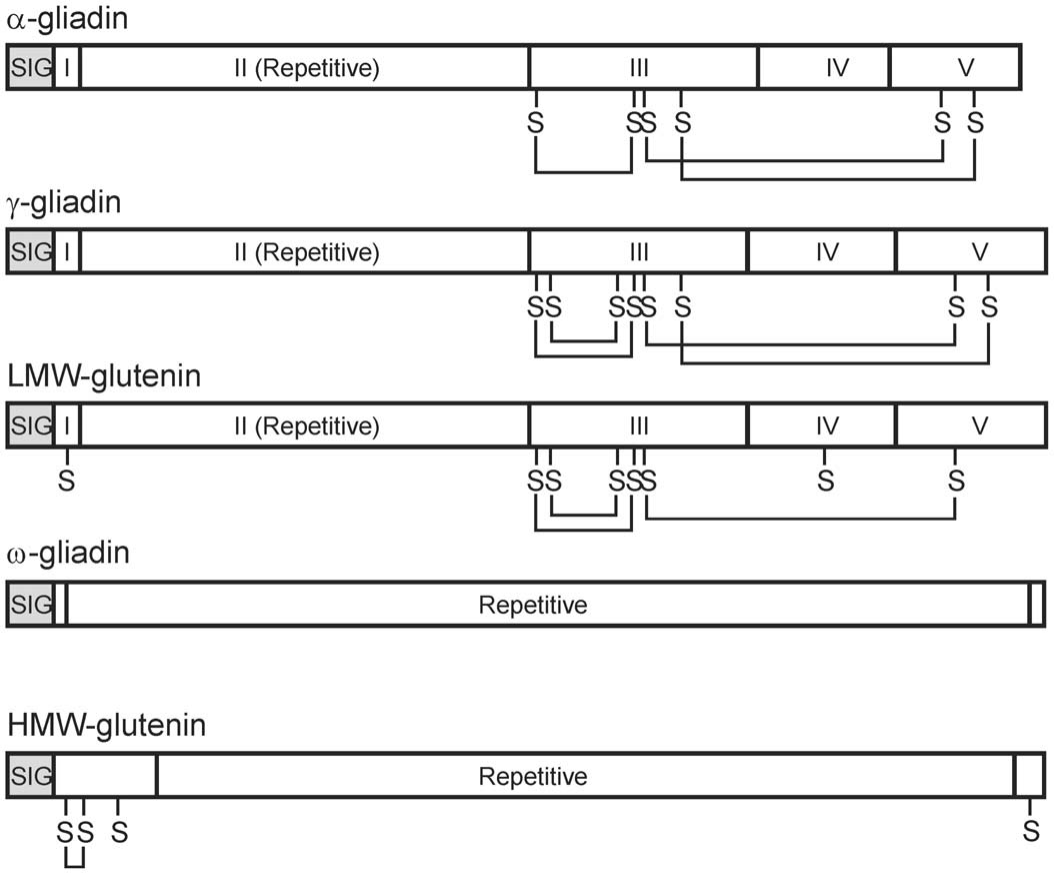
General Structure of the wheat prolamins. The general structure of the wheat prolamin classes is diagrammed showing the main sequence domains, conserved cysteine residues (S), and intramolecular disulfide bonds (lines connecting Ss). The signal peptides (SIG) are shaded. The mature polypeptide sequence of the α- and γ-gliadins and LMW-glutenins are composed of five sections: (I) a short non-repetitive peptide, (II) the repetitive domain composed of variations of short motifs, (III) a non-repetitive region containing most of the cysteine residues, (IV) a glutamine-rich domain, and (V) the C-terminal non-repetitive domain containing at least one cysteine residue. The ω-gliadins usually have no cysteines and therefore no disulfide bonds. The disulfide bonds are taken from references: α-gliadins [15], γ-gliadins [16], and the HMW- and LMW-glutenins [17].

The wheat seed proteins are predominantly prolamins – polypeptides high in glutamine and proline amino acid residues, and whose primary structure includes a region of repeats composed of variations on distinct motifs for each prolamin class. The wheat prolamins have historically been divided into glutenin (high- and low-molecular-weight; HMW and LMW) and gliadin (α-, γ-, and ω-gliadin) types dependent on whether they form polymers or exist mainly as monomers, respectively. The general structure of these five classes of wheat prolamins are shown in Figure 1.

Similar seed proteins are found in other members of the Triticeae tribe, including barley (*Hordeum*) – considered evolutionarily distant from wheat within the Triticeae. Comparison of wheat and barley prolamin genes has led to suggestions of orthologous pairings; i.e., the HMW-glutenins and D-hordeins, the LMW-glutenins and the B-hordeins, the ω-gliadins and the C-hordeins, and the γ-gliadins and γ-hordeins. Similar homoeologous chromosome 1 locations further support these orthologous pairings. The wheat α-gliadins are found on wheat chromosome 6 and related genes exist in many other Triticeae, but not barley, and are believed to have arisen as a translocation of one or more ancestral gliadin genes from chromosome 1 to chromosome 6 [1].

**Figure 2:**
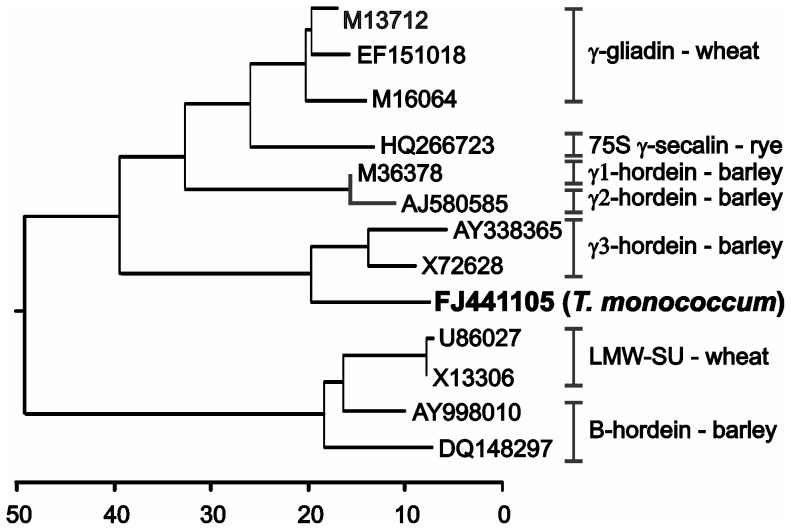
Phylogenetic tree of Triticeae γ-type prolamins compared to a *T. monococcum* cDNA. Derived amino acid sequences, minus repetitive domains (see text), were aligned with Clustal V. The root of the tree was using an α-gliadin as an outgroup. Genbank accession numbers identify each sequence. Classifications of related genes are shown with brackets and names on the right of the figure.

The present study reports on a fortuitous discovery of a novel wheat prolamin not previously distinguished from the large gliadin and LMW-glutenin sequence families. The single prolamin-like gene sequence from *Brachypodium distachyon* [18] was used by blastn analysis to find which Triticeae prolamin genes were the most similar, and thus possibly the most related to the origins of the Triticeae prolamins. The closest matches were to wheat LMW-glutenins and barley B-hordeins, at approximately e^−18^ and e^−12^ respectively, followed by matches to other prolamins. Although the similarity results were insufficient to address the original issue, there was one curious finding. Among the best of the hundreds of wheat LMW-gluten and barley B-hordein matches was a single partial *T. monococcum* cDNA (FJ441105) annotated as a γ-gliadin and several γ3-hordein sequences from *Hordeum vulgare and H. chilense*, e.g., M72628 and AY338365. A comparison of these three sequences to Triticeae γ-type prolamins of wheat, barley, and rye, along with the LMW-glutenin/B-hordein orthologous prolamins is shown in the phylogenetic tree in Figure 2. As expected, the barley B-hordeins are most closely related to the wheat LMW-glutenins, and the γ-gliadins, γ-hordeins, and γ-secalins branch together. However, the *T. monococcum* and barley γ3-hordein sequences cluster as a separate branch from the branch containing γ-prolamins from barley, rye, and wheat.

Estimations of sequence relatedness can also be obtained from comparative blast results. When Genbank is interrogated with a γ-gliadin coding sequence (AF234646), annotated wheat γ-gliadin sequences are returned with blastn expectation values of e = 0 to e^−178^, and the barley γ1- and γ2-hordein sequences returned with e^−116^ and e^−82^, respectively (not shown) – indicating a close relationship between the wheat and barley γ-prolamins. In contrast, γ3-hordein sequences only match to the wheat γ-gliadins starting at e^−16^. To compare, another class of wheat prolamins belonging to the gliadin-superfamily are the LMW-glutenins whose matches with the γ-gliadin probe begin at about e^−34^. A similar indication of significant divergence between the γ1- and γ2-hordein sequences compared to the γ3-hordein sequence is that when either the γ1- or γ2-hordein sequences are interrogated to Genbank, the identified γ3-hordein sequences do not appear until e^−16^– e^−15^, and after two other classes of prolamins, the B- and C-hordeins (not shown). These results suggest that the γ1- and γ2-hordein sequences are members of the same barley prolamin family, with γ3-hordein being a distinct class of prolamins. Until now, no orthologous wheat sequences to a barley γ3-hordein have been reported, so it has not been clear if the barley γ3-hordein is a unique development in barley versus other Triticeae.

The comparisons shown in Figure 2 indicate that the *T. monococcum* cDNA is related to the γ3-hordein, but what about polyploid wheats? Based on these results, an in-depth search was carried out on available wheat genomic and EST sequences to confirm the existence of this novel wheat prolamin class, to obtain a full-length sequence not available from the single *T. monococcum* sequence, and determine gene copy numbers.

### EST and Genomic DNAs

The publically available EST resource was screened for similarities to the barley γ3-hordein and *T. monococcum* sequences initially focusing on ESTs from cv Chinese Spring. Seventeen ESTs were found to match and these ESTs assembled into two closely related 3′ prolamin sequences, neither of which encoded a full-length protein (not shown). Two additional ESTs matched the 5′ portion of the barley γ3-hordein, but did not overlap with the other 15 ESTs – presumably due to a gap in the available sequence. ESTs from other cultivars were too few to generate full-length coding sequences although they confirmed the general structure of the coding regions (not shown).

In an attempt to recover the entire coding regions, next-generation high-throughput whole genomic sequences were searched for DNA similar to the barley γ3-hordein and *T. monococcum* sequences by probing two Roche 454 sequence collections; i.e., a 5× coverage of the wheat hexaploid cv Chinese Spring and a 3× coverage of the wheat D genome ancestor, the diploid *Ae. tauschii*. A total of 69 Chinese Spring and 10 *Ae. tauschii* 454 matching reads were identified. Given an average matching 454 read length of 400 bp and a γ3-hordein coding region plus immediate flanking regions totaling about 1000 bp, it is possible to estimate the number of gene copies represented by these numbers of 454 reads – assuming random distribution of 454 sequences across the genomes. For both DNA sources, the crude estimate is approximately 1–2 copies per genome. This estimate was confirmed after assembling the reads; i.e., the hexaploid Chinese Spring assembly contained four distinguishable sequences and the diploid *Ae. tauschii* assembly two distinct sequences (not shown). The individual Chinese Spring 454 reads and ESTs were reassembled into two full-length intact coding regions and a third full-length sequence with two tandem in-frame premature stop codons – all three sequences being similar to the barley γ3-hordein sequence (all consensus Chinese Spring and *Ae. tauschii* sequences are available in File S1). These results explain why only two different sequences were found among the Chinese Spring ESTs; i.e., the mRNA of one Chinese Spring gene is likely unstable due to the premature stop codons.

From *Ae. tauschii* there was a single apparently intact coding sequence that matched one of the Chinese Spring consensus sequences. There were also *Ae. tauschii* reads whose consensus sequence matched the fourth Chinese Spring sequence and is a pseudogene with one in-frame stop codon, one single base deletion, and one 11 base deletion - both the latter two leading to frame shifts. This pseudogene is present in both Chinese Spring and *Ae. tauschii* and therefore existed in the *Ae. tauschii* gene pool before the hybridization creating hexaploid bread wheats.

**Figure 3:**
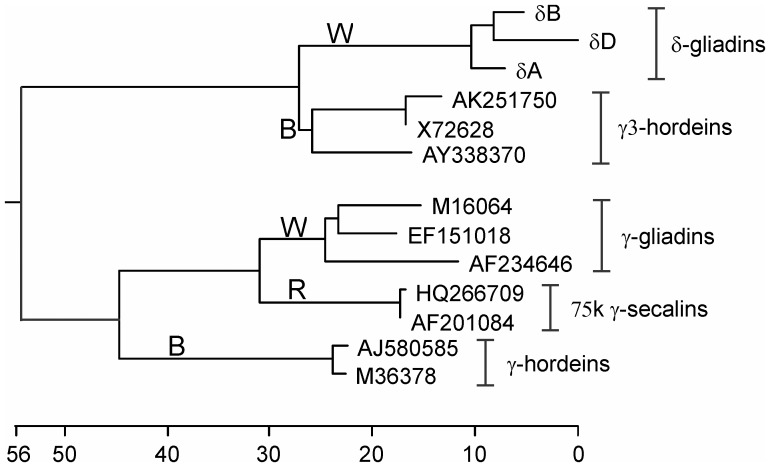
Phylogenetic tree of γ-type Triticeae prolamins and δ-gliadins. Derived amino acid sequences are aligned with Clustal V as in Figure 1. Genbank accession numbers identify each sequence. Classifications of related genes are shown with brackets and names on the right of the figure. The root of the tree was using an α-gliadin sequence as an outgroup. Branches containing sequences from only one Triticeae species are shown by capital letters on or near those branches: wheat (W), barley (B), rye (R).

Since there has been no previous recognition of wheat sequences orthologous to the γ3-hordeins, and since the sequences are distinctive enough to indicate a separate class of wheat prolamins (more below), we propose a new nomenclature for this distinct branch separate from the γ-gliadins. To be consistent with wheat nomenclature, they will henceforth be referred to as δ-gliadins and recognized as orthologous to the barley γ3-gliadins. The three full-length Chinese Spring derived amino acid sequences were compared with γ-type prolamins and the resulting phylogenetic tree is shown in Figure 3. The δ-gliadins are the three topmost sequences in the tree and are labeled with their genome origin (determined below); e.g., δA represents the δ-gliadin from the A-genome.

Two main branches are shown in Figure 3. The upper branch contains the δ-gliadins and γ3-hordeins, and the lower branch the related γ-prolamins from wheat, rye, and barley. Since the repetitive domains of the prolamins were included in the analysis, and since these repetitive domains (Domain II in Figure 1) change more rapidly than non-repetitive sequences, and might skew comparative results, further analyses were carried out and are shown in Figure S1. If the repetitive domains of the polypeptides are removed, the resulting phylogenetic tree of amino acid sequences confirms Figure 3 (Figure S1A). Similarily, a comparison of DNA sequences (for those prolamin classes with available flanking DNA sequences) from 400 bp upstream of the start codon to 100 bp downstream of the stop codon, minus the repetitive regions, shows the same separate branches for the δ-gliadin/γ3-hordein and the remaining γ-type prolamins (Figure S1B).

Insufficient genomic or EST sequences are available from other Triticeae to determine if there are δ-gliadin orthologs in other Triticeae besides *T. aestivum*, *T. monococcum*, barley and *Ae. tauschii*. However, it is likely such orthologs will be found as more Triticeae are subjected to deeper sequencing.

### Genome Assignments

Assignments of the three distinct δ-gliadin sequences to specific genomes was by comparison to sequences of known genome source (Figure S2). Although the *T. monococcum* A^m^ genome is not the direct ancestor of the hexaploid A genome (thought to be *T. urartu*), it is close enough to distinguish among the hexaploid A, B, and D genomes. The partial cDNA sequence from *T. monococcum* (FJ441105) mismatched over its sequence by 6, 17, and 31 bases when compared to the three Chinese Spring δ-gliadin sequences (not shown). Therefore, the closest matching δ-gliadin sequence is assigned to the A genome and is referred to as the δA gene.

The D-genome sequence was assigned by matching to 454 DNA sequences from the partial *Ae. tauschii* δ-gliadin sequence. Over the 847 bases of aligned positions, one of the hexaploid Chinese Spring sequences differed from the diploid *Ae. tauschii* δ-gliadin sequence by only two individual bases and a single glutamine codon (CAA) in a polyglutamine-encoding region (not shown). This second δ-gliadin of Chinese Spring is therefore assigned to the D genome (δD).

The exact ancestor of the hexaploid B genome is unknown, but is believed to be related to *Ae. speltoides*. However, no relevant sequences are yet known from such a B-genome relative. It is tentatively assumed, by elimination, that the third sequence represents the B-genome δ-gliadin (δB).

### Structure of δ-gliadins

**Figure 4:**
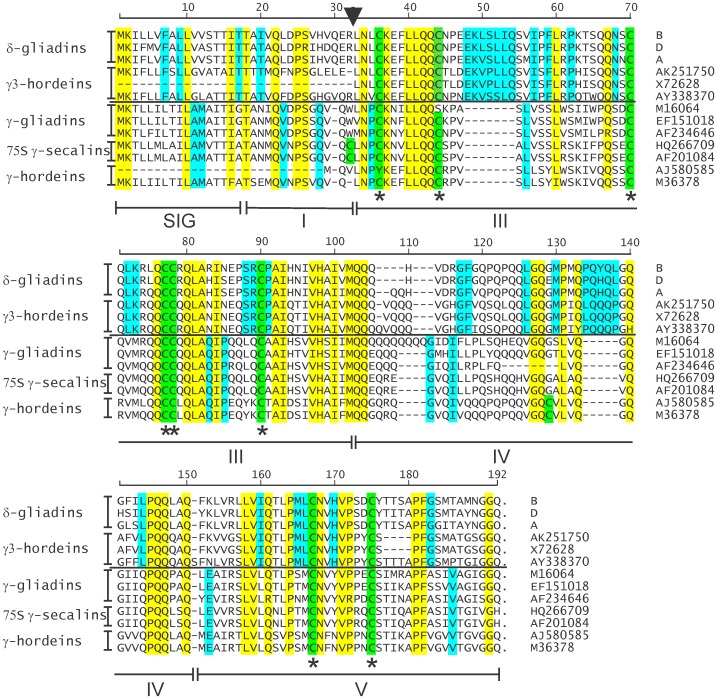
Alignment of δ-gliadins with γ-type prolamins. The derived amino acid sequences for δ-gliadins and barley γ3-hordeins are aligned with wheat, barley, and rye γ-prolamins, but without the repetitive domains (position of the repetitive domain indicated by the arrowhead). The vertical order is the same as in Figure 3. Residue positions common to all aligned sequences are shaded with yellow. Residue positions shared only by the upper or lower initial branches of Figure 3 are shaded with blue. Horizontal lines separate sequences from the two initial branches of Figure 3. Accession numbers of previously reported sequences are given on the right, and classifications by prolamin type is given to the left. Cysteine residues are shaded in green and conserved cysteine positions are indicated by asterisks below the alignments. Domains similar to Figure 1 are indicated below the alignments.

The amino acid sequences of the three different Chinese Spring δ-gliadins are aligned in Figure 4 with the same prolamins (in order) as in the phylogenetic tree of Figure 3. The repetitive domains are not included in this alignment since attempting to align the fast changing repetitive domains among the prolamins can lead to false alignments due to prevalence of proline and glutamine residues. Although the Chinese Spring δA gene contains two premature stop codons, Figure 4 shows the amino acid sequence encoded by the non-repetitive portion of the gene. The general structure of the wheat δ-gliadins is similar to that shown in Figure 1 for the γ-gliadins. A SIG domain is the signal peptide cleaved during protein processing. Domains II and IV are glutamine-rich, with domain II composed on variations of a repeat motif and domain IV is glutamine-rich (glutamines in 15 of 43 residues for δA in Figure 4) without a clear repeat structure. Domains I, III, and IV are non-repetitive, with domains III and IV containing the conserved cysteine positions (shaded green in Figure 4) that can form four intramolecular disulfide bonds assuming similar bond patterns to other gliadin classes.

There are known examples of gliadins with odd numbers of cysteines that could form intermolecular bonds, e.g. γ-gliadins [19], [20], γ-hordein [8], and both 75S γ-secalins of Figures 3 and 4. However, the conserved even number of cysteines in these δ-gliadins indicates there are no cysteines likely available for intermolecular disulfide bonds that could serve as gluten polymer chain terminators [1] – at least for this particular germplasm (Chinese Spring).

In comparison to the general conservation of the eight cysteine residues with γ-type gliadins, the three δ-gliadins shown in Figure 4 share 21 amino acid residues and two insertions with γ3-hordeins but not the other γ-type prolamins. The distinctive residue pattern between the δ-gliadin/γ3-hordein orthologs (above the line in Figure 4) compared to the γ-type gliadins from wheat, rye, and barley (below the line in Figure 4) are emphasized by yellow shading of residues common to all the sequences in Figure 4 and the blue shading for residues conserved in either the δ-gliadin/γ3-hordeins or the γ-type prolamins.

### Repetitive Domains

**Figure 5:**
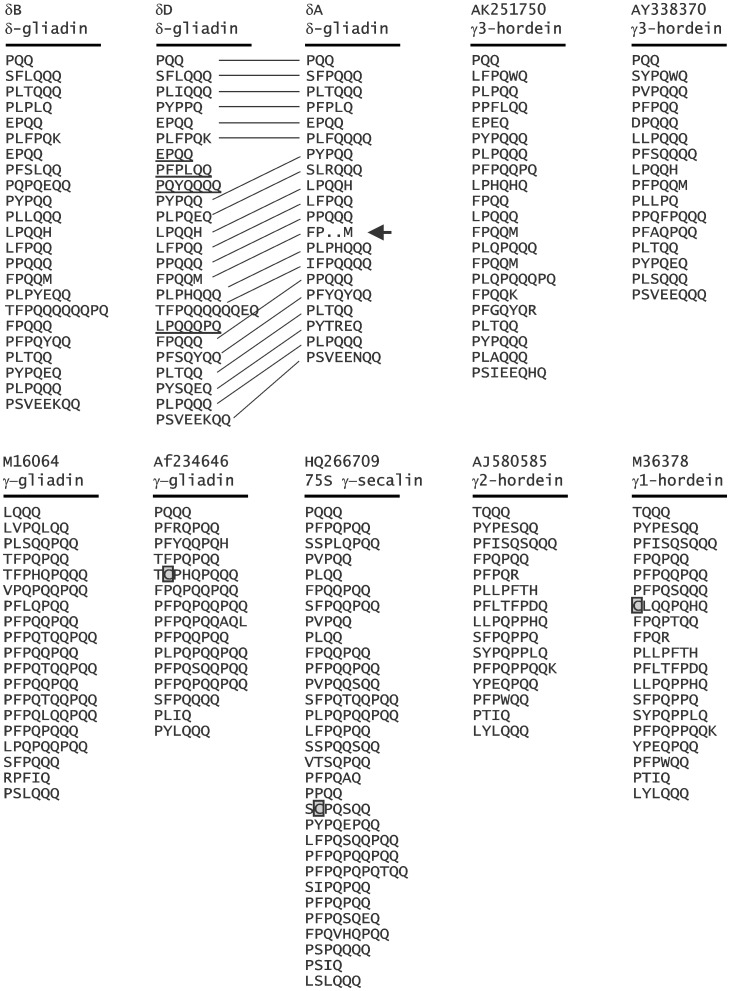
Prolamin repetitive domains. The repetitive domains of several Triticeae prolamins are shown with repeats arrayed vertically. The sequences from which the repetitive domains originate are identified by Genbank accession numbers and membership in different prolamin classes. Lines connecting repeat motifs in δD and δA indicate conserved repeats. Underlined repeats in δD are repeat differences with δA. Cysteine residues are boxed. The arrow indicates the repeat motif that is FPQQM in wheat hexaploid cv Recital, but FP.M in cv Chinese Spring.

To compare the repetitive domains of the δ-gliadins to the different γ-prolamin types, the respective repetitive domains are shown with repeat motifs arrayed vertically in Figure 5. For some prolamins, such as the HMW-glutenins and ω-gliadins, the repeats are sufficiently regular to simplify repeat divisions in such a display. However, for other prolamins the repeats are to a varying degree more irregular. In such cases there is no obvious best method of defining a repetitive motif. Prolamin repeat motifs tend to be rich in proline and both single and short runs of glutamine. For the current analysis we define repeat motifs as the most common pattern within that specific prolamin – with most repeats beginning with a proline (P) and ending with several glutamines (Q). As seen in Figure 5, the repeat motif pattern for both the wheat δ-gliadins and barley γ3-hordeins is based on the pattern P-L/F-P-Q_2–3_– with many variants. Such variations are commonly caused by single base changes that convert a proline or glutamine codon to a codon for another amino acid (such as CAA → AAA, CAG → GAG, or CCG → TCG). This δ-gliadin repeat pattern is similar to the P-F/Y-P-Q_3–5_ pattern of the α-gliadins [21] and P-F-P/S-Q_2–5_ pattern of the LMW-glutenins [22]. In contrast, the overall motif pattern for the wheat γ-gliadins, rye γ-secalins, and barley γ1- and γ2-hordeins is based more on P-F-(P-Q_1–2_)-P-Q-Q. Again pointing to differences between the δ-gliadin/γ3-hordeins and the γ-gliadins/γ-hordeins and which, along with phylogenetic trees, blast comparisons, and amino acid sequence comparisons shown earlier provide support for considering the δ-gliadins/γ3-hordeins as a distinct class of Triticeae prolamins.

It is assumed, from comparing sample members of the prolamin families, that the repetitive domain evolves mainly both by single amino acid changes and deletions and/or duplications of sections of the repetitive domain. These deletions/duplications are evidenced by differences in the order of the repeat motifs. For example, in Figure 5, lines connect suggested conserved repeat motifs between the δD and δA repeat domains. Underlined motifs in the δD repetitive domain are motifs missing in the δA repetitive domain. Whether the differences are due to deletions or duplications cannot be ascertained from examining only a few sequences. Note that the arrow in Figure 5 indicates a repeat containing two tandem premature stops in the Chinese Spring δA gene.

In addition to the occasional odd number of cysteines in gliadin in the non-repetitive portion of prolamins (caused through an amino acid residue change), some repetitive domains also contain cysteines; e.g., seen boxed in Figure 5 for a γ-gliadin (AF234646), a 75S γ-secalin (HQ266709), and a γ-hordein (M36378). Thus far, the δ-gliadins contain only the eight conserved cysteine residues in domains III and V (Figures 1 and 4) and are assumed to be monomeric.

### δ-gliadin Genes as Part of a Complex Prolamin Chromosomal Region

The γ-gliadins, ω-gliadins, and LMW-glutenins are linked on the short arm of the group 1 chromosomes and were initially reported to be part of the complex *Gli-1* wheat locus (Payne et al. 1984), but more detailed mapping found recombinants that separate at least some of the LMW-glutenins into additional loci (reviewed in [23]).

**Figure 6:**
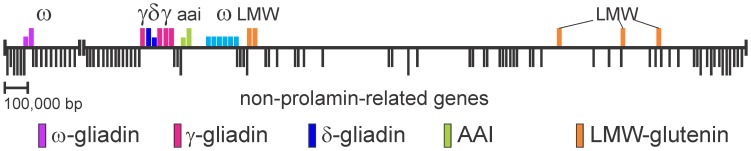
δ-gliadin gene chromosome location. A 3.1 Mb region of the *Ae. tauschii* 1D chromosome covered by 28 overlapping BACs in two contigs was sequenced as indicated by the horizontal line. Prolamin and closely related AAI (alpha-amylase-inhibitor) genes are indicated above the line with colors identifying prolamin types. Longer colored lines indicate apparently intake reading frames while shorter colored lines indicate pseudogenes or gene fragments. Annotated non-prolamin genes are indicated by black vertical lines below the region sequence. Longer black vertical lines indicate syntenic genes among the known grass genomes.

The order of annotated genes within two BAC contigs spanning a 3.l Mb region of *Ae. tauschii* chromosome 1D (assembled from 28 overlapping BAC clones; Genbank JX295577) is diagrammed in Figure 6. Positions of prolamin and α-amylase inhibitor genes (distantly related to the prolamins) are indicated by colored vertical lines above the horizontal line representing the 3.1 Mb chromosome region. Shorter colored lines indicate pseudogenes or gene fragments. Black vertical lines below the horizontal line indicate non-prolamin genes. Longer black lines are genes whose synteny is conserved in other grasses (*Brachypodium*, rice, sorghum) and form the basis of orienting the two contigs. The BAC library was originally screened with γ-gliadin and LMW-glutenin probes. Since no additional BACs were found with either of the two probe genes, it is likely no additional prolamin genes are in the contig gap and that this region represents the entire *Ae. tauschii* prolamin gene cluster for δ-gliadins, γ-gliadins, ω-gliadins, and LMW-glutenins.

The two *Ae. tauschii* δ-gliadin sequences (blue vertical lines in Figure 6) include one full-length coding region and a second sequence which is a pseudogene. These two sequences match the two D-genome δ-gliadins from the 454 genomic assemblies and apparently represent the entire family of δ-gliadin sequences in the D-genome. These two sequences also are flanked by γ-gliadin sequences, while the ω-gliadin sequences are in two clusters bracketing the δ- and γ-gliadins and two alpha-amylase-inhibitor sequences, and the five LMW-glutenin sequences widely separated and interspersed with numerous genes, none of which are related to prolamins. These results show a non-uniform arrangement of wheat prolamin genes – the order of genes resulting from a complex history of tandem gene duplication/deletions, and segmental duplication/deletions.

### Expression of δ-gliadin Genes

It has already be noted above that Chinese Spring ESTs exist for two of the three full-length δ-gliadin genes, with the third sequence not found in Chinese Spring ESTs due to the two in-frame premature stop codons in the δA gene. To determine if the δ-gliadin genes were expressed in other cultivars, searches were carried out with the Genbank EST databases. ESTs matching to the δ-gliadin sequences were found for five different hexaploid wheat cultivars. Only two of those have sufficient available seed ESTs to make useful counts of matching ESTs for a single gene, i.e., the hexaploid cultivars Chinese Spring and Recital. A total of 17 and 14 δ-gliadin ESTs were identified for Chinese Spring and Recital, respectively. For Chinese Spring, four ESTs matched δB and 13 ESTs matched δD. No ESTs matched δA. In contrast, for cv Recital, no ESTs matched δB, but five ESTs matched δD and nine matched δA – implying differential expression of the three δ-gliadin orthologs between the two cultivars. To estimate if the observed distribution of ESTs across the three genomes could be by chance, the total assigned ESTs from the two cultivars were subjected to a Chi-square goodness-of-fit test. The result was p = .0076, supporting rejection of the possibility the distribution was by chance. Since the number of cv Recital ESTs is small, it cannot be determined if the Recital δB gliadin gene is inactive/missing or simply expressing at a much lower rate than orthologous genes.

### δ-gliadin Orthologous Genes and Expression in Other Triticeae

Whether the δ-gliadins are represented in other Triticeae by single genes per genome, as they are in wheat, can only be currently addressed by available resources in barley. Although there are only two different γ3-hordein nucleotide sequences from *H. vulgare* in Genbank, with one full-length coding sequence, the extensive barley ESTs resource can be screened. The barley ESTs are mainly from three *H. vulgare* cultivars, i.e., Barke, Morex, and Optic. ESTs from each of these three barley cultivars were identified and assembled separately for each cultivar. A total of 140 γ3-gliadin ESTs were found for cv Barke, 60 in cv Morex, and 28 ESTs in cv Optic. For each of the three cultivars, the ESTs assembled into a single contig containing a complete coding region - with no evidence of more than one sequence – agreeing with one active δ-gliadin gene per genome in wheat. In Figure S3, the three derived barley γ3-hordein amino acid sequences are aligned with the only two available *H. vulgare* γ3-hordein sequences and with two *H. chilense* sequences. The cv Barke polypeptide is identical to *H. vulgare* X72628 (cv hor2ca) and AK251750 (cv Haruna Nijo) except for one residue difference in the latter. The Morex and Optic polypeptide sequences are identical to each other, but different from the other three polypepetides with duplication/deletion of two repetitive motifs and extension of a polyglutamine run from three to six residues.

### Conclusions

The barley γ3-hordein prolamin has a previously unrecognized ortholog in wheat that is here designated as a δ-gliadin. The δ-gliadin/γ3 hordeins occur as a single active gene per genome in hexaploid wheats, diploid *Ae. tauschii*, and barley, although different δ-gliadin gene orthologs may be inactive in different hexaploid cultivars. A δ-gliadin pseudogene occurs in the D genome of hexaploid Chinese Spring and diploid *Ae. tauschii*, and both the intact δ-gliadin and pseudogene of *Ae. tauschii* are located with the complex chromosomal region that also contains the γ-gliadin, ω-gliadin, and LMW-glutenin genes.

## Supporting Information

Figure S1 **Phylogenetic analyses of Triticeae γ-type prolamins.** Sequence alignments of Triticeae prolamins are used to generate phylogenetic trees suggesting evolutionary relationships among γ-type prolamins. The repetitive domains are removed from the alignments to avoid distortions caused by misalignments of the differentially changing tandem repetitive motifs compared to non-repetitive sequences. Alignments are by Clustal V. A) Encoded polypeptide sequences are aligned. B) DNA sequences from 400 bp upstream of the start codon to 100 bp downstream of the stop for each prolamin gene are aligned. No γ-hordein gene flanking DNA is available. For both frames, classes of Triticeae prolamins are indicated to the right of sequence identifications.
(TIF)

Figure S2 **Genome assignments of δ-gliadin sequences.** The three wheat δ-gliadin gene sequences from cv Chinese Spring are aligned with the cDNA from *T. monococcum* (A^m^ genome) and the single δ-gliadin genomic sequence from the diploid D-genome ancestor *Ae. tauschii*. Alignments were carried out with Clustal V.
(TIF)

Figure S3 **Barley γ3-hordein sequences.** Barley ESTs from cvs Barke, Morex, and Optic were separately assembled and the derived full-length derived amino acids sequences are aligned. Hv = *H. vulgare*, Hc = *H. chilense.* The single amino acid residue (R) difference among *H. vulgare* sequences at position 128 is shaded in blue. Differences in the *H. chilense* sequences compare to *H. vulgare* are shaded in yellow.
(TIF)

File S1 **Fasta file of δ-gliadins and γ3-hordeins.** Consensus assembled δ-gliadin DNA sequences from hexaploid wheat Chinese Spring and *Ae. tauschii* plus barley γ3-hordeins assembled from cultivars Barke, Morex, and Optic are given in fasta format along with derived protein sequences.
(TXT)
