# Supplementary figures and images for: A New Class of Wheat Gliadin Genes and Proteins
Source: PLoS One. 2012 Dec 20;7(12):e52139. doi: 10.1371/journal.pone.0052139 (PMC3527421; doi:10.1371/journal.pone.0052139)

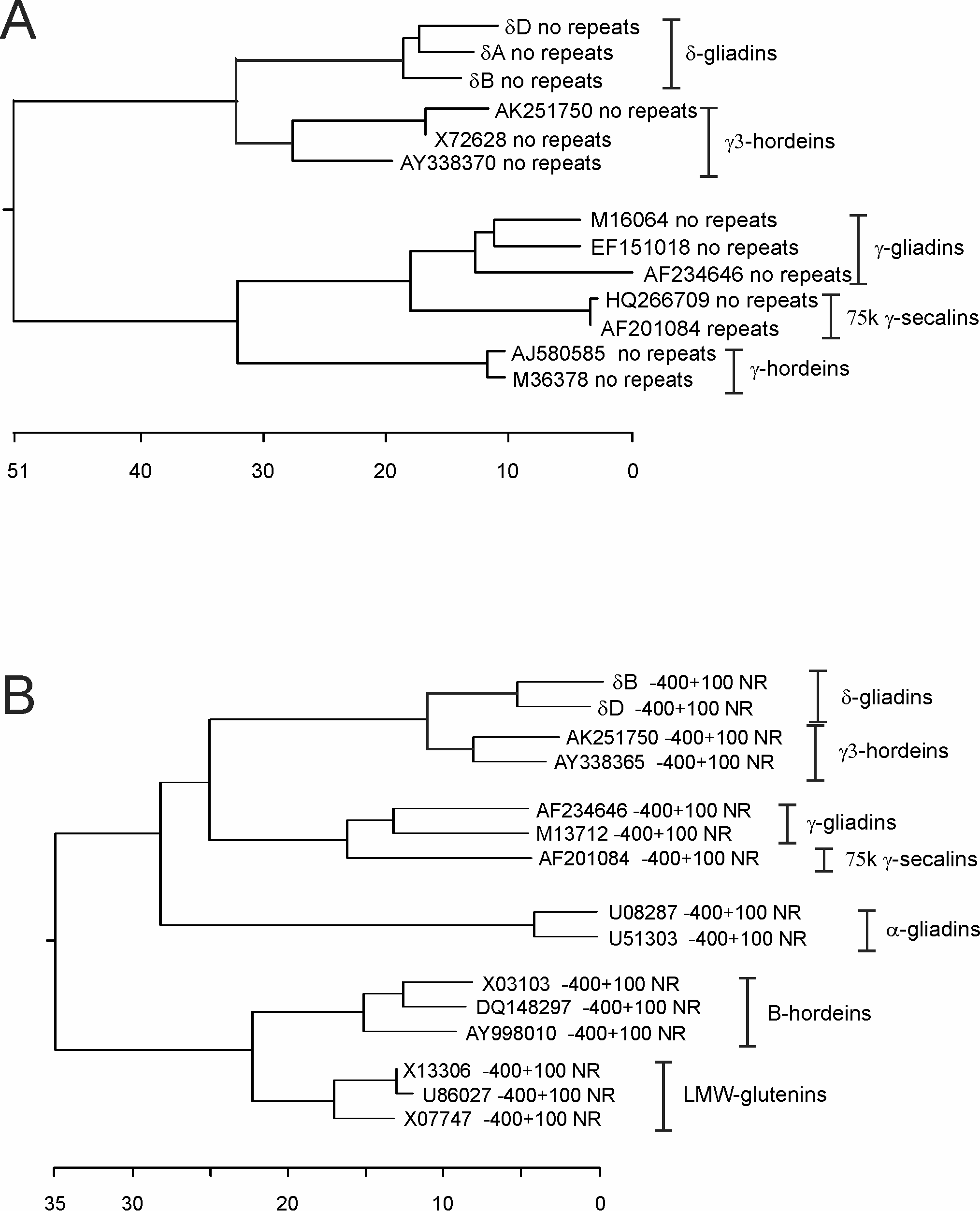

Supplement: Figure S1 — Phylogenetic analyses of Triticeae γ-type prolamins. Sequence alignments of Triticeae prolamins are used to generate phylogenetic trees suggesting evolutionary relationships among γ-type prolamins. The repetitive domains are removed from the alignments to avoid distortions caused by misalignments of the differentially changing tandem repetitive motifs compared to non-repetitive sequences. Alignments are by Clustal V. A) Encoded polypeptide sequences are aligned. B) DNA sequences from 400 bp upstream of the start codon to 100 bp downstream of the stop for each prolamin gene are aligned. No γ-hordein gene flanking DNA is available. For both frames, classes of Triticeae prolamins are indicated to the right of sequence identifications. (TIF) [file pone.0052139.s001.tif]

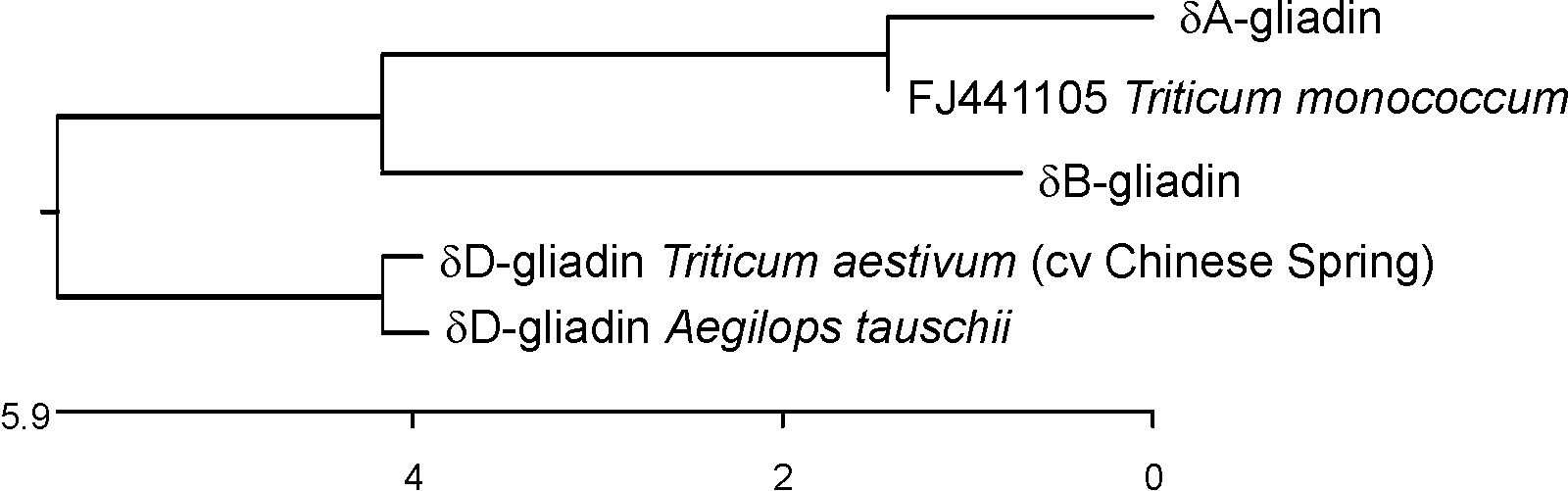

Supplement: Figure S2 — Genome assignments of δ-gliadin sequences. The three wheat δ-gliadin gene sequences from cv Chinese Spring are aligned with the cDNA from T. monococcum (Am genome) and the single δ-gliadin genomic sequence from the diploid D-genome ancestor Ae. tauschii. Alignments were carried out with Clustal V. (TIF) [file pone.0052139.s002.tif]
